# Supplementary material for: Light-Seq: light-directed in situ barcoding of biomolecules in fixed cells and tissues for spatially indexed sequencing
Source: Nat Methods. 2022 Oct 10;19(11):1393–402. doi: 10.1038/s41592-022-01604-1 (PMC9636025; doi:10.1038/s41592-022-01604-1)
Supplement: Supplementary file 1 — Supplementary Information contains Supplementary Figs. 1–3, Tables 1–6, Notes 1–4 and references. [file 41592_2022_1604_MOESM1_ESM.pdf]

nature portfolio

https://doi.org/10.1038/s41592-022-01604-1

Supplementary information

Light-Seq: light-directed in situ barcoding of biomolecules in fixed cells and tissues for spatially indexed sequencing

In the format provided by the authors and unedited

Supplementary Information

Light-Seq: Light-directed *in situ* barcoding of biomolecules in fixed cells and tissues for spatially indexed sequencing

Jocelyn Y. Kishi[1,2,*,✉], Ninning Liu[1,*], Emma R. West[3,4,*], Kuanwei Sheng[1], Jack J. Jordanides[1], Matthew Serrata[1], Constance L. Cepko[3,4,5,✉], Sinem K. Saka[1,2,6,✉] and Peng Yin[1,2,✉]

[1] Wyss Institute for Biologically Inspired Engineering, Harvard University, Boston, Massachusetts 02115, USA.
[2] Department of Systems Biology, Harvard Medical School, Boston, Massachusetts 02115, USA.
[3] Department of Genetics, Blavatnik Institute, Harvard Medical School, Boston, Massachusetts 02115, USA.
[4] Howard Hughes Medical Institute, Chevy Chase, Maryland 20815, USA.
[5] Department of Ophthalmology, Harvard Medical School, Boston, Massachusetts 02115, USA.
[6] Genome Biology Unit, European Molecular Biology Laboratory (EMBL), 69117 Heidelberg, Germany.

* These authors contributed equally.
✉ Correspondence should be addressed to jocelyn.y.kishi@gmail.com, sinem.saka@embl.de, py@hms.harvard.edu, or cepko@genetics.med.harvard.edu.

Supplementary Information contains:

Supplementary Figure 1. ROI selection on microscope (HEK cells).
Supplementary Figure 2. ROI selection on microscope (3T3 cells).
Supplementary Figure 3. Strand design schematics.

Supplementary Table 1. Estimated UMI counts per area for human HEK cells and mouse 3T3 cells.
Supplementary Table 2. Top transcripts detected in human HEK cells and mouse 3T3 cells.
Supplementary Table 3. Estimated UMI per area and estimated cell numbers for mouse retina by retinal layer.
Supplementary Table 4. Barcoded area and UMI counts of TH^+ amacrine cells.
Supplementary Table 5. Cost estimates for barcoding reagents.
Supplementary Table 6. Sequences of DNA oligos used in the experiments.
Supplementary Table 7. List of SABER-FISH probe sets used for RNA-FISH validation experiments (**provided as a separate spreadsheet**).

Supplementary Note 1. Design choices to limit background during reverse transcription.
Supplementary Note 2. Background blocking.
Supplementary Note 3. Intronic mapping for retina tissue experiments.
Supplementary Note 4. Instructions for calibrating, focusing, optimizing, and selecting ROIs for DMD illumination, and important considerations for resolution and experimental design.

Supplementary References

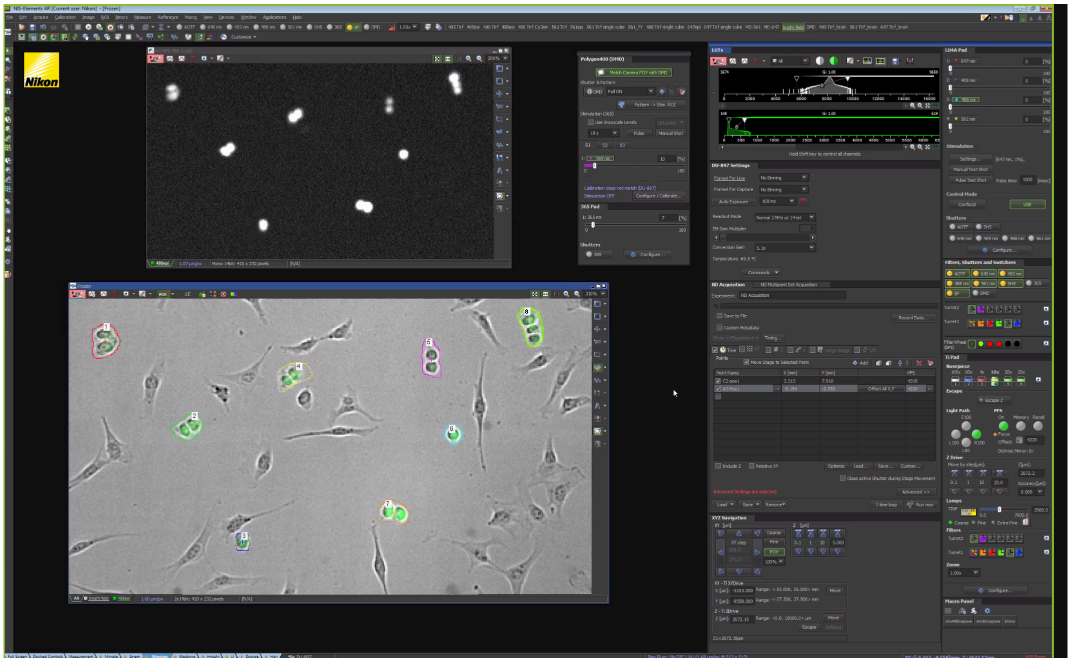

Supplementary Figure 1. ROI selection on microscope (HEK cells). Screenshot of Nikon software showing ROIs chosen based on Brightfield + GFP image overlay for HEK-eGFP cell barcoding for cells shown in **Fig. 3d-f**.

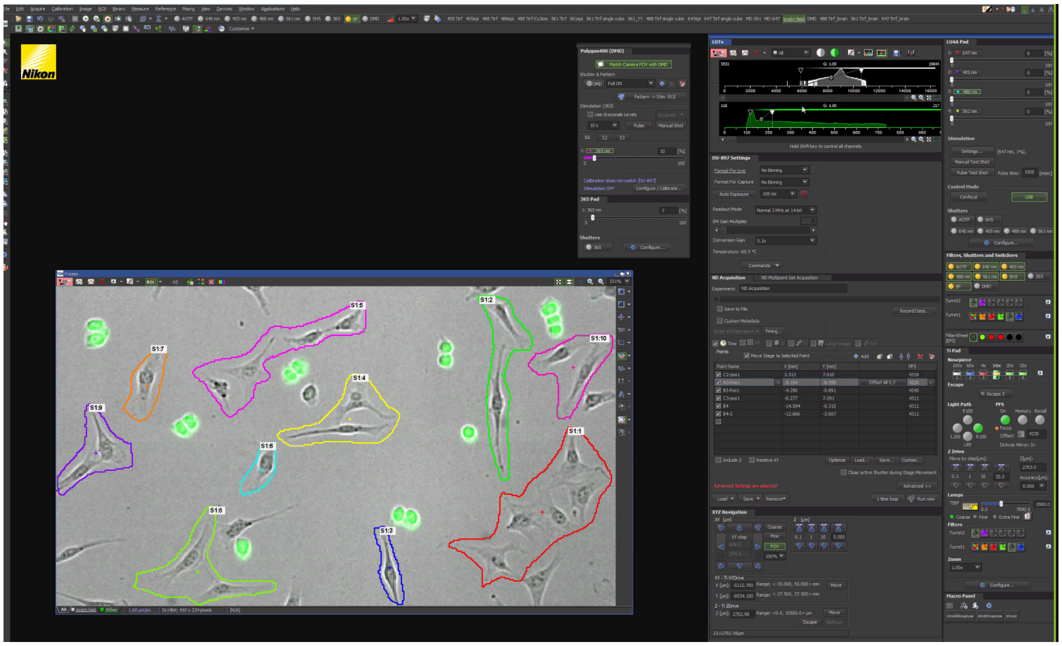

Supplementary Figure 2. ROI selection on microscope (3T3 cells). Screenshot of Nikon software showing ROIs chosen based on Brightfield + GFP image overlay for 3T3 cell barcoding for cells shown in **Fig. 3d-f**.

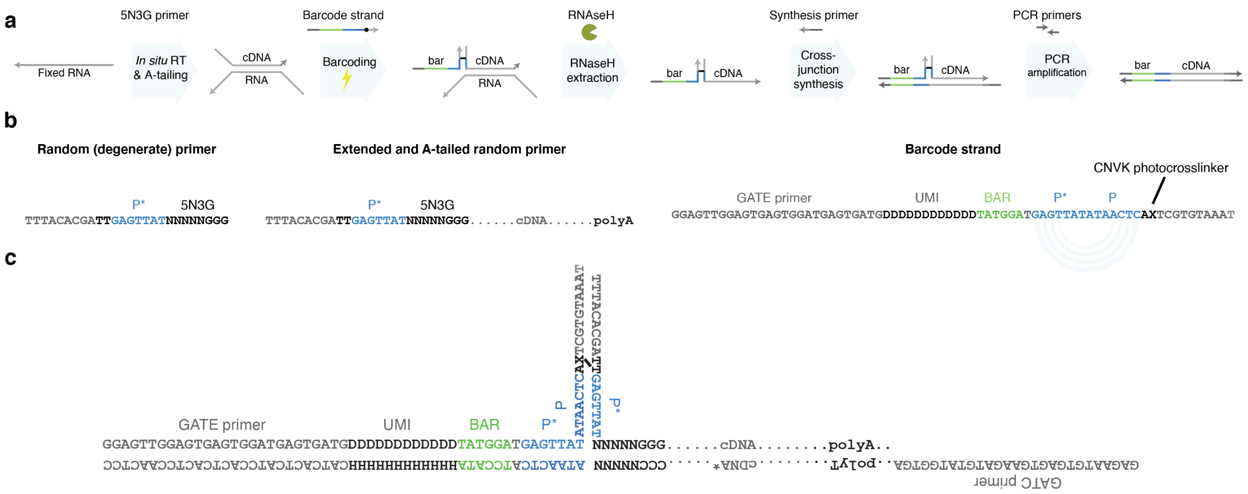

Supplementary Figure 3. Strand design schematics. **(a)** Schematic depicting barcoding strand design. **(b)** Random primer, extended primer, and barcode strand design. **(c)** Example junction sequence design and the final concatenated output strand.

Supplementary Table 1. Estimated UMI counts per area for human HEK cells and mouse 3T3 cells. To obtain the number of UMIs per unit area, we first estimated the total of the cell area (in pixels) that was subject to UV illumination for each barcode based on microscopy images of the ROIs. We calculated the total illuminated cellular area by segmenting the cells to discard any potential non-cell areas that were included in the ROIs by the manual selection. Hence barcoded area in pixels is estimated via a segmentation mask. We then convert this to μm^2 by multiplying it with the pixel size in microns (pixel scaling 0.6556 µm x 0.6556 µm per pixel), dividing the total area by 100 μm^2 to find how many unit areas it corresponds to, and finally dividing the UMI counts (correctly barcoded and uniquely mapped reads for the corresponding sample) by the total number of illuminated cell or tissue area units. We note that although roughly the same numbers of HEK and 3T3 cells were barcoded, the total barcoded cellular area for 3T3 cells is 2.3-3-fold larger than the HEK cell area. Half of each replicate sample was amplified, and after library preparation all replicates were pooled together on a single HiSeq lane and sequenced. One single replicate (Replicate 2 below) was also separately run on a single HiSeq lane for deeper sequencing (Deeper Seq.).

	Human 1	Human 2	Human 3	Mouse 1	Mouse 2	Mouse 3
Total Barcoded Area (pixels)	15,755	18,932	17,139	40,319	44,846	52,283
UMI's / unit area (10 µm × 10 µm)	1,436	2,234<br><br>3,328 (Deeper Seq.)	2,207	982	1,392<br><br>2,029 (Deeper Seq.)	1,135

Supplementary Table 2. Top transcripts detected in human HEK cells and mouse 3T3 cells.
Top 50 are shown. Full list is provided as **Source Data Table 1**.

**Human HEK**			**Mouse 3T3**		
**Gene ID**	**Gene name**	**Gene type**	**Gene ID**	**Gene name**	**Gene type**
ENSG00000210082.2	MT-RNR2	Mt_rRNA	ENSMUSG00000064339.1	mt-Rnr2	Mt_rRNA
ENSG00000211459.2	MT-RNR1	Mt_rRNA	ENSMUSG00000064370.1	*mt-Cytb*	protein_coding
ENSG00000198886.2	*MT-ND4*	protein_coding	ENSMUSG00000064341.1	*mt-Nd1*	protein_coding
ENSG00000210100.1	MT-TI	Mt_tRNA	ENSMUSG00000064344.1	mt-Tm	Mt_tRNA
ENSG00000198938.2	*MT-CO3*	protein_coding	ENSMUSG00000064351.1	*mt-Co1*	protein_coding
ENSG00000212907.2	*MT-ND4L*	protein_coding	ENSMUSG00000064345.1	*mt-Nd2*	protein_coding
ENSG00000198727.2	*MT-CYB*	protein_coding	ENSMUSG00000065701.3	Rny1	misc_RNA
ENSG00000198786.2	*MT-ND5*	protein_coding	ENSMUSG00000086324.9	Gm15564	lncRNA
ENSG00000198804.2	*MT-CO1*	protein_coding	ENSMUSG00000064367.1	*mt-Nd5*	protein_coding
ENSG00000198840.2	*MT-ND3*	protein_coding	ENSMUSG00000064368.1	*mt-Nd6*	protein_coding
ENSG00000198899.2	*MT-ATP6*	protein_coding	ENSMUSG00000029580.15	*Actb*	protein_coding
ENSG00000198712.1	*MT-CO2*	protein_coding	ENSMUSG00002076940.1	ENSMUSG	miRNA
ENSG00000228253.1	*MT-ATP8*	protein_coding	ENSMUSG00000064363.1	*mt-Nd4*	protein_coding
ENSG00000198763.3	*MT-ND2*	protein_coding	ENSMUSG00000064338.1	mt-Tv	Mt_tRNA
ENSG00000198888.2	*MT-ND1*	protein_coding	ENSMUSG00000001506.11	*Col1a1*	protein_coding
ENSG00000198695.2	*MT-ND6*	protein_coding	ENSMUSG00000064336.1	mt-Tf	Mt_tRNA
ENSG00000210176.1	MT-TH	Mt_tRNA	ENSMUSG00000068220.7	*Lgals1*	protein_coding
ENSG00000210049.1	MT-TF	Mt_tRNA	ENSMUSG00000064340.1	mt-Tl1	Mt_tRNA
ENSG00000283907.1	LLNLF-96A1.1	lncRNA	ENSMUSG00000035783.10	*Acta2*	protein_coding
ENSG00000225840.2	AC010970.2	processed_pseudogene	ENSMUSG00000023944.15	*Hsp90ab1*	protein_coding
ENSG00000210112.1	MT-TM	Mt_tRNA	ENSMUSG00000034994.11	*Eef2*	protein_coding
ENSG00000210184.1	MT-TS2	Mt_tRNA	ENSMUSG00000001525.11	*Tubb5*	protein_coding
ENSG00000256222.3	*MTRNR2L3*	protein_coding	ENSMUSG00000031375.18	*Bgn*	protein_coding
ENSG00000167658.16	*EEF2*	protein_coding	ENSMUSG00000026193.16	*Fn1*	protein_coding
ENSG00000184009.13	*ACTG1*	protein_coding	ENSMUSG00000001131.12	*Timp1*	protein_coding
ENSG00000283657.1	RP11-646I19.1	unprocessed_pseudogene	ENSMUSG00000035202.9	*Lars2*	protein_coding
ENSG00000168298.7	*H1-4*	protein_coding	ENSMUSG00000078812.11	*Eif5a*	protein_coding
ENSG00000075624.17	*ACTB*	protein_coding	ENSMUSG00000040856.18	*Dlk1*	protein_coding
ENSG00000210191.1	MT-TL2	Mt_tRNA	ENSMUSG00000060802.9	*B2m*	protein_coding
ENSG00000183779.7	*ZNF703*	protein_coding	ENSMUSG00000026728.10	*Vim*	protein_coding
ENSG00000096384.20	*HSP90AB1*	protein_coding	ENSMUSG00000000753.16	*Serpinf1*	protein_coding
ENSG00000248527.1	MTATP6P1	unprocessed_pseudogene	ENSMUSG00000025362.7	*Rps26*	protein_coding
ENSG00000247627.2	*MTND4P12*	processed_pseudogene	ENSMUSG00000029661.17	*Col1a2*	protein_coding
ENSG00000109971.14	*HSPA8*	protein_coding	ENSMUSG00000003814.9	*Calr*	protein_coding
ENSG00000198034.11	*RPS4X*	protein_coding	ENSMUSG00000025132.14	*Arhgdia*	protein_coding
ENSG00000249780.1	RP11-352E6.2	processed_pseudogene	ENSMUSG00000063457.15	*Rps15*	protein_coding
ENSG00000108518.8	*PFN1*	protein_coding	ENSMUSG00000056201.9	*Cfl1*	protein_coding
ENSG00000196230.14	*TUBB*	protein_coding	ENSMUSG00000007892.9	*Rplp1*	protein_coding
ENSG00000167978.17	*SRRM2*	protein_coding	ENSMUSG00000031328.17	*Flna*	protein_coding
ENSG00000175130.7	*MARCKSL1*	protein_coding	ENSMUSG00000018293.5	*Pfn1*	protein_coding
ENSG00000274997.2	*H2AC12*	protein_coding	ENSMUSG00000064080.13	*Fbln2*	protein_coding
ENSG00000034510.6	*TMSB10*	protein_coding	ENSMUSG00000018593.14	*Sparc*	protein_coding
ENSG00000177600.9	*RPLP2*	protein_coding	ENSMUSG00000070436.13	*Serpinh1*	protein_coding
ENSG00000274641.2	*H2BC17*	protein_coding	ENSMUSG00000068566.14	*Myadm*	protein_coding
ENSG00000286522.2	*H3C2*	protein_coding	ENSMUSG00000037742.15	*Eef1a1*	protein_coding
ENSG00000110955.9	*ATP5F1B*	protein_coding	ENSMUSG00000002602.17	*Axl*	protein_coding
ENSG00000166165.13	*CKB*	protein_coding	ENSMUSG00000029860.17	*Zyx*	protein_coding
ENSG00000074800.16	*ENO1*	protein_coding	ENSMUSG00000032399.9	*Rpl4*	protein_coding
ENSG00000197903.8	*H2BC12*	protein_coding	ENSMUSG00000026547.16	*Tagln2*	protein_coding
ENSG00000210144.1	MT-TY	Mt_tRNA	ENSMUSG00000024529.15	*Lox*	protein_coding

Supplementary Table 3. Estimated UMI per area and estimated cell numbers for mouse retina by retinal layer.

Barcoded area in pixels was calculated from the binary image used for photomasks. Pixel size is 1.6 μm/pixel.

	**Section 1**	**Section 2**	**Section 3**	**Section 4**
ONL barcoded area (pixels)	14,091	13,142	10,669	8,594
BCL barcoded area (pixels)	6,380	6,798	5,969	5,365
GCL barcoded area (pixels)	5,448	5,744	4,195	4,381
ONL UMIs per unit area (10 μm x 10 μm)	2,220	2,148	1,603	1,410
BCL UMIs per unit area (10 μm x 10 μm)	1,186	1,197	1,287	1,472
GCL UMIs per unit area (10 μm x 10 μm)	5,628	4,537	6,057	5,221
Number of cells ONL	1,221	1,309	1,062	856
Number of cells BCL	309	330	290	261
Number of cells GCL	101	106	77	81
Total barcoded area as % of total tissue section area	10.8	10.5	10.0	7.6

Supplementary Table 4. Barcoded area and UMI counts of TH^+ amacrine cells.
Barcoded area in pixels was calculated from the binary image used for photomasks. Pixel size is 1.6 μm/pixel.

**TH+ Amacrine Cells**	**Section 1**	**Section 2**	**Section 3**	**Section 4**	**Section 5**
Total barcoded area (pixels)	227	327	217	296	118
UMI counts	48,978	87,449	42,507	46,245	30,317
Number of cells	7	8	6	7	4
UMI per unit area (10 μm x 10 μm)	8,428	10,446	7,651	6,102	10,036
UMI per cell (TH^+ DAC)	6,996	10,931	7,084	6,606	7,579

Supplementary Table 5. Cost estimates for barcoding reagents.

The cost of performing reverse transcription, A-tailing, three rounds of barcoding, displacement, cross-junction synthesis, and bulk PCR is estimated to be around $34.50 based on the amount of each material used for these steps (for a buffer volume of 50 µl/sample on a single well in an 18-well µ-slide). The reagent amounts will scale with the incubation volume for the desired sample/chamber format. For most items, we included online non-discounted commercial list prices as of March 26, 2022 (underlined). For oligos, the item with no public list price (KK2502), and the item that has been discontinued (GE27-2051-01), we listed our paid price. We also note that the cost of the optional fluorophore modifications (Cy5, Cy3, and Fluorescein) added significantly to the cost of the barcode strands (which we ordered in bulk, at 1 µmole scale). We note that the list prices for the reagents may vary depending on the desired order size and academic discounts. Each additional barcode round is projected to cost <$1.50. Note that this estimate does not include the standard costs of the imaging chambers, sample prep (e.g. fixation), library prep, or sequencing.

Reagent name	Vendor	Catalog #	List price or paid price	Amount	Amount in µl	Amount used for barcoding one 50 µl well	Cost per 50 µl well
PBS - Phosphate-Buffered Saline (10X) pH 7.4, RNase-free	Invitrogen	AM9625	$76.75	1 L	1,000,000	300	$0.02
Ultrapure water	Invitrogen	10977023	$207.00	10 x 500 ml	5,000,000	1924.315	$0.08
Thermo Scientific™ Maxima H Minus Reverse Transcriptase (200 U/µL) (includes 5X buffer)	Thermo Scientific	FEREP0753	$844.00	4 x 10,000 units	200	2	$8.44
RNaseOUT™ Recombinant Ribonuclease Inhibitor	Invitrogen	10777019	$208.00	5k units, 40 U/µl	125	3	$4.99
Triton™ X-100	Sigma Aldrich	T8787-50ML	$31.20	50 ml	50,000	0.25	$0.00
Terminal Transferase - 2,500 units	NEB	M0315L	$301.00	2,500 units, 20k units/ml	125	2.5	$6.02
2′,3′-Dideoxyadenosine 5′-Triphosphate, 100 mM solution (ddATP)	Sigma Aldrich	GE27-2051-01	$309.00	4 µmol of 100 mM	40	0.0125	$0.10
Deoxynucleotide (dNTP) Solution Set - 25 µmol each at 100 mM (just need dATP)	NEB	N0446S	$180.00	25 µmol each of 100 mM	250	0.5	$0.36
Deoxynucleotide (dNTP) Solution Mix - 8 µmol at 10mM each	NEB	N0447S	$66.00	8 µmol of 10 mM	800	2.3	$0.19
5M NaCl	Invitrogen	AM9760G	$45.38	100 ml	100,000	225	$0.10
Formamide (Deionized)	Invitrogen	AM9342	$119.00	500 ml	500,000	780	$0.19
Salmon-sperm DNA	Thermo Fisher	AM9680	$304.00	10 tubes, 1 ml each	10,000	30	$0.91
Dextran Sulfate 50% solution	EMD	S4030	$175.00	100 ml	100,000	30	$0.05
Bst DNA Polymerase, Large Fragment - 8,000 units (includes 10XTP and 100mM MgSO4)	NEB	M0275L	$296.00	8,000 U, 8,000 unit/ml	1,000	8	$2.37
RNase H - 1,250 units	NEB	M0297L	$298.00	1,250 U, 5000 units/ml	250	3.375	$4.02
HiFi HotStart DNA Polymerase, Kapa Biosystems	Roche	KK2502	$302.45	250 U (enzyme), 1 U/µl	250	3.2	$3.87
SYBR™ Green I Nucleic Acid Gel Stain - 10,000X concentrate in DMSO	Invitrogen	S7563	$372.00	10,000X conc	500	0.008	$0.01
TWEEN® 20	Sigma Aldrich	P9416-50ML	$24.20	50 ml	50,000	3	$0.00
10 x 2 ml IDTE pH 7.5 (1X TE Solution)	IDT	11-01-02-02	$18.00	20 ml	20,000	12.519	$0.01
RT primer (10 µM)	IDT	250 nmol scale, HPLC purified	$69.05	206 µl of 100 µM	2,060	5	$0.17
Barcode 1 strand (10 µM), Cy5 labeled	Gene Link	1 µmol scale, PAGE purified, priority shipping	$1,265.20	21.7 nmol (217 µl 100 µM)	2,170	1.25	$0.73
Barcode 2 strand (10 µM), Cy3 labeled	Gene Link	1 µmol scale, PAGE purified, priority shipping	$1,265.20	15.2 nmol (152 µl 100 µM)	1,520	1.25	$1.04

Barcode 3 strand (10 µM), Fluorescein labeled	Gene Link	1 µmol scale, PAGE purified, priority shipping	$869.25	18.1 nmol (181 µl 100 µM)	1,810	1.25	$0.60
Cross-junction synthesis primer (10 µM)	IDT	100 nmol scale, HPLC purified	$50.95	176 µl of 100 µM	1,760	0.16	$0.00
PCR primer 1 (10 µM)	IDT	250 nmol scale, HPLC purified	$69.60	288 µl of 100 µM	2,880	4.8	$0.12
PCR primer 2 (10 µM)	IDT	250 nmol scale, HPLC purified	$69.60	319 µl of 100µM	3,190	4.8	$0.10
						**Total:**	**$34.50**

Supplementary Table 6. Sequences of DNA oligos used in the experiments.
Barcode strands with CNVK were ordered from Gene Link at 1 μmol scale, all others were ordered from IDT at 100 nmol or 250 nmol scale. All oligo stocks were kept in IDTE buffer at -20°C. Working dilutions of 10 μM in IDTE were typically made for frequently used oligos. We note that the custom index primer is compatible with HiSeq, but for NovaSeq, unique i7 indices were needed for de-convolution due to inefficient i5 index sequencing.

Sequence name	Purpose	Sequence	Purification	Vendor
GATE.D12.B1	Barcode sequence 1 - Cy5 labeled barcode strand.	GGAGTTGGAGTGAGTGGATGAGTGATGDDDDDDD DDDDDTATGGATGAGTTATATAACTCA[cnvK]TCGT GTAAAT[Cy5-3]	PAGE	Gene Link
GATE.D12.B2	Barcode sequence 2 - Cy3 labeled barcode strand.	GGAGTTGGAGTGAGTGGATGAGTGATGDDDDDDD DDDDDGTTAGGTGAGTTATATAACTCA[cnvK]TCGT GTAAAT[Cy3-3]	PAGE	Gene Link
GATE.D12.B3	Barcode sequence 3 - Fluorescein (FITC) labeled barcode strand.	GGAGTTGGAGTGAGTGGATGAGTGATGDDDDDDD DDDDDAGGGTATGAGTTATATAACTCA[cnvK]TCGT GTAAAT[Fl-3]	PAGE	Gene Link
RT.5N.3G	Primer for reverse transcription.	TTTACACGATTGAGTTATNNNNNGGG	HPLC	IDT
GATC.20T	Primer for cross-junction synthesis.	GAGAATGTGAGTGAAGATGTATGGTGATTTTTTTTT TTTTTTTTTTT	HPLC	IDT
GATE	PCR primer 1.	GGAGTTGGAGTGAGTGGATGAGTGATG	HPLC	IDT
GATC	PCR primer 2.	GAGAATGTGAGTGAAGATGTATGGTGA	HPLC	IDT
S502.GATE	Primer for library prep - i5 (barcode) side.	AATGATACGGCGACCACCGAGATCTACACCTCTCT ATCGCCGGAGTTGGAGTGAGTGGATGAGTGATG	HPLC	IDT
S503.GATE	Primer for library prep - i5 (barcode) side.	AATGATACGGCGACCACCGAGATCTACACTATCCT CTCGCCGGAGTTGGAGTGAGTGGATGAGTGATG	HPLC	IDT
S505.GATE	Primer for library prep - i5 (barcode) side.	AATGATACGGCGACCACCGAGATCTACACGTAAGG AGCGCCGGAGTTGGAGTGAGTGGATGAGTGATG	HPLC	IDT
S506.GATE	Primer for library prep - i5 (barcode) side.	AATGATACGGCGACCACCGAGATCTACACACTGCA TACGCCGGAGTTGGAGTGAGTGGATGAGTGATG	HPLC	IDT
S507.GATE	Primer for library prep - i5 (barcode) side.	AATGATACGGCGACCACCGAGATCTACACAAGGAG TACGCCGGAGTTGGAGTGAGTGGATGAGTGATG	HPLC	IDT
S508.GATE	Primer for library prep - i5 (barcode) side.	AATGATACGGCGACCACCGAGATCTACACCTAAGC CTCGCCGGAGTTGGAGTGAGTGGATGAGTGATG	HPLC	IDT
Next.N701	(Normal) primer for library prep - i7 side.	CAAGCAGAAGACGGCATACGAGATTCGCCTTAGTC TCGTGGGCTCGG	HPLC	IDT
Next.N702	(Normal) primer for library prep - i7 side.	CAAGCAGAAGACGGCATACGAGATCTAGTACGGTC TCGTGGGCTCGG	HPLC	IDT
Next.N703	(Normal) primer for library prep - i7 side.	CAAGCAGAAGACGGCATACGAGATTTCTGCCTGTC TCGTGGGCTCGG	HPLC	IDT
Next.N704	(Normal) primer for library prep - i7 side.	CAAGCAGAAGACGGCATACGAGATGCTCAGGAGT CTCGTGGGCTCGG	HPLC	IDT
Next.N705	(Normal) primer for library prep - i7 side.	CAAGCAGAAGACGGCATACGAGATAGGAGTCCGT CTCGTGGGCTCGG	HPLC	IDT
Next.N706	(Normal) primer for library prep - i7 side.	CAAGCAGAAGACGGCATACGAGATCATGCCTAGTC TCGTGGGCTCGG	HPLC	IDT
P5.GATE	Custom Read 1 Primer - required for sequencing of amplicons.	CGCCGGAGTTGGAGTGAGTGGATGAGTGATG	HPLC	IDT
GATE*.P5*	Custom i5 index primer - required for some Illumina sequencers (see caption).	CATCACTCATCCACTCACTCCAACTCCGGCG	HPLC	IDT
D0*.7N.30A.bio	Biotinylated strand for *in vitro* glass slide labeling.	TTTACACGATTGAGTTATNNNNNNNAAAAAAAAAA AAAAAAAAAAAAAAAAAAAA/3Bio/	Desalted (STD)	IDT

Supplementary Note 1. Design choices to limit background during reverse transcription.

The well-known non-templated addition of bases onto the 3' end of strands by Moloney murine leukemia virus (MMLV)-type reverse transcriptases (including Maxima H minus which we use here) has been utilized in standard reverse transcription (RT) reactions as a way to achieve cDNA synthesis and template switching in a one pot reaction[1,2]. By incorporating a template switching oligo (TSO), typically ending in 3 RNA G bases, double-stranded DNA products can be formed (see below)[1,3].

Standard RT: no fixation, diffusive, one-pot cDNA synthesis and template switching to create PCR amplifiable products.

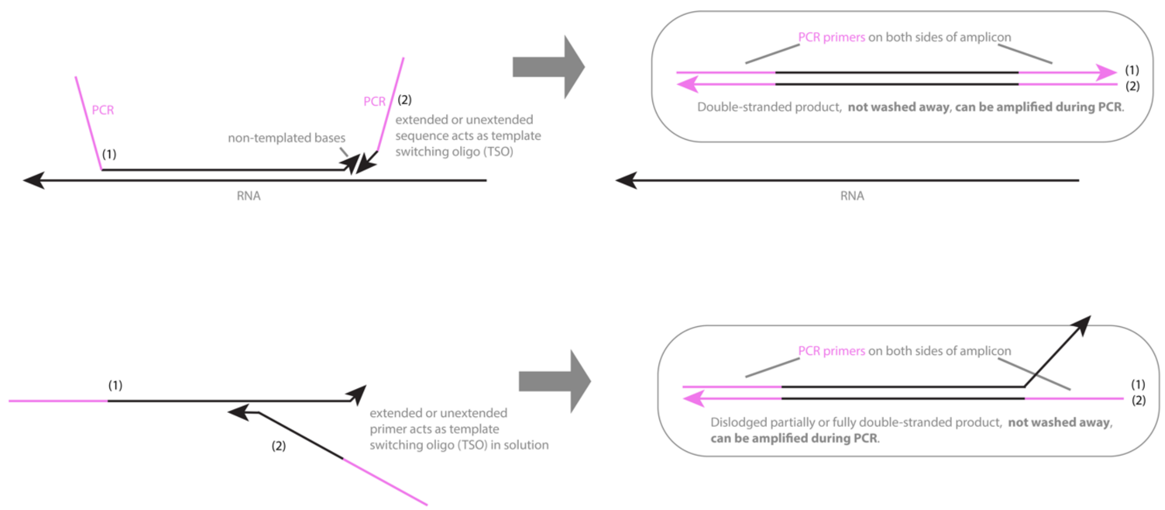

Due to the affinity to G bases, it is possible that our random 5N3G reverse transcription primer could itself act as a template switching oligo (TSO) during reverse transcription: However, in contrast to that and other standard RT protocols[4], we have several major design differences that we believe limit the potential for these and other types of template switching reactions to affect our sequencing reads in a meaningful way:

(1) We perform extensive washing after RT. Because samples are fixed, we are able to perform extensive stringent washing to help remove background products that have formed and become dislodged during the RT step (further explained below). There are also extensive subsequent washes during barcoding.

(2) Since we barcode on the 5' end of our cDNA reads, our RT primer does not contain a PCR primer. Thus, in order to have background products generated during RT get amplified in PCR, they would have to stay lodged within the sample, get barcoded, and then become dislodged specifically during the RNase H treatment.

(3) Because samples and RNAs are fixed in place, rather than diffuse as in standard RT reactions, the likelihood of a sequence "jumping" to another RNA is low, unless it has already become dislodged from its original RNA sequence (further explained below).

(4) As we use RNase H to release cDNA sequences *in situ*, the dislodging of sequences is expected to be specific to DNA-RNA hybrids, so any single- or double-stranded DNA background products formed and stuck *in situ* should remain in place.

Below, we examine potential unwanted reactions that could occur during RT due to the strand displacing and non-templated addition properties of the reverse transcriptase (Scenarios 1-5). We also performed analysis on our paired-end data to show that we see no strong evidence of read 1 (R1) and read 2 (R2) sequences mapping to different RNA transcripts.

Scenario 1: strand-displacing reverse transcriptase displaces extended cDNA sequences

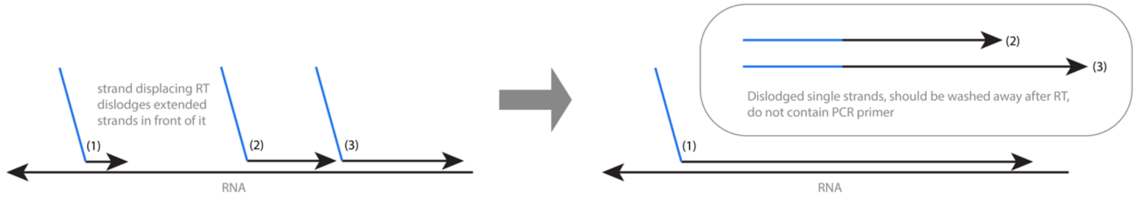

Scenario 1: Due to the internal priming of RNA sequences, the high strand displacement activity of the reverse transcriptase enzyme, and the repeated temperature cycling to anneal primers, we expect that some extended cDNA sequences could become dislodged from their original RNA molecules *in situ*. However, unlike in standard RT reactions, we perform extensive stringent washing immediately following RT to try to remove any of these that might form. Also, unlike standard RT reactions, these sequences also do not contain PCR primers so they could not be exponentially amplified during PCR unless at least several more steps happen.

Scenario 2: excess RT primer or dislodged extended sequences act as template switching oligos (TSOs)

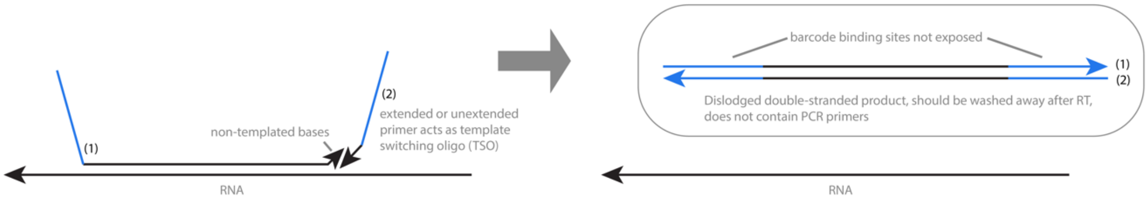

Scenario 2: Due to the non-templated addition of bases by reverse transcriptase, and particularly because we use the 5N3G RT primer, secondary reactions such as the one shown above could potentially occur. The RT primer, or even extended and dislodged cDNA sequences formed in Scenario 1, could act as TSOs to form double-stranded products. Fortunately, these double-stranded products would not have exposed barcode binding sites (shown in blue), nor do they have PCR primers on either end of them. Thus, multiple additional steps would have to happen in order for these reads to become exponentially amplified in PCR.

Scenario 3: extended cDNA sequence jumps directly to another RNA template (unlikely if RNA fixed in place)

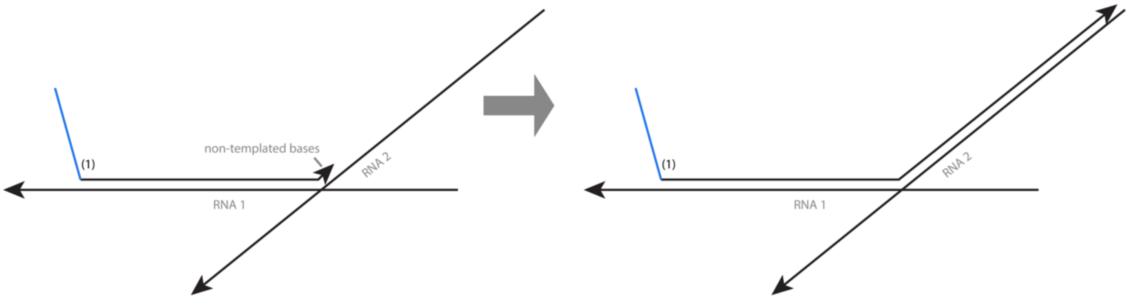

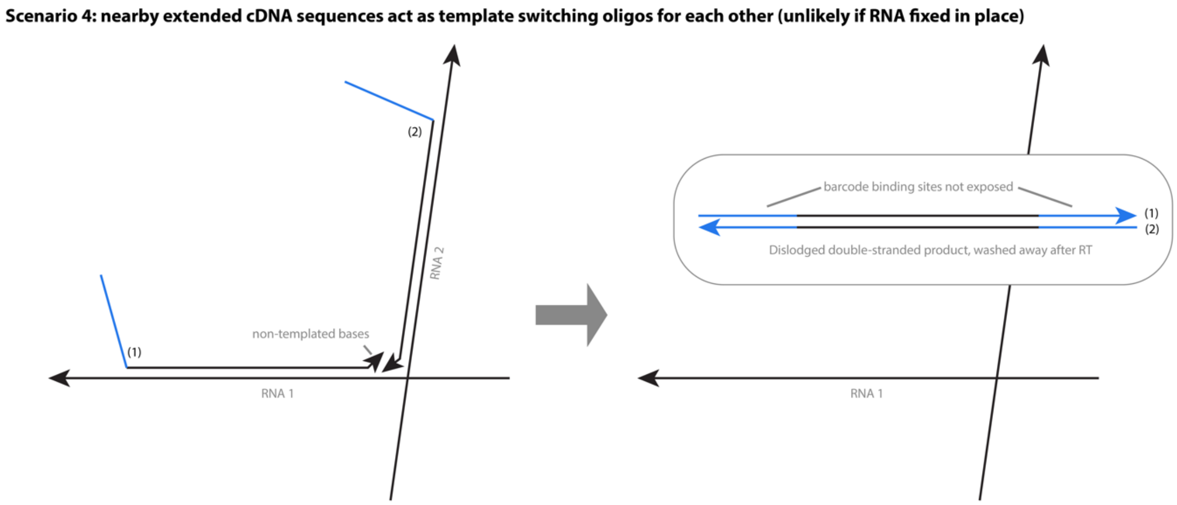

Scenarios 3 and 4: It is also conceivable that cDNA sequences could directly jump to other RNA sequences or other cDNA sequences still attached to their RNA molecules. However, because the RNAs are fixed in place (rather than being diffusive, as in standard RT), we expect this to be extremely unlikely.

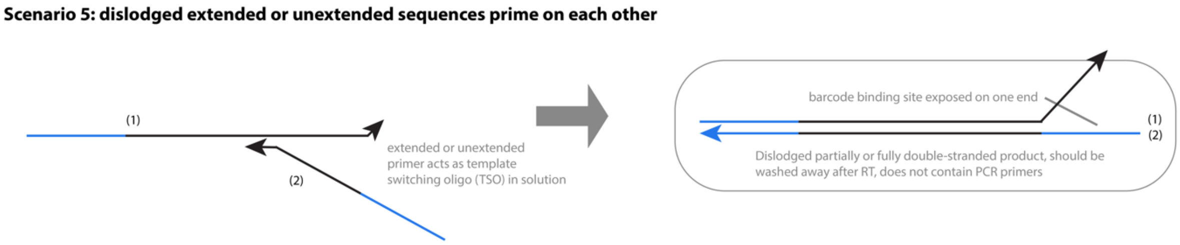

Scenario 5: After single-stranded sequences are dislodged (Scenario 1), they have the potential to prime on, or serve as a template for, other diffusing single-stranded sequences such as unextended primers or other extended cDNA sequences. However, because they are not attached to RNAs, they should be washed away after the RT step, do not contain PCR primers, and if they got stuck nonspecifically in the sample should not be displaced by RNase H as there is no RNA-DNA hybrid. It is, however, possible that a variation of Scenario 5 happens such that template switching occurs but is not complete, so that some part of one of the sequences remains bound *in situ* to RNA. If this occurred, and one of the barcode binding sites was exposed and subsequently barcoded, then we could potentially end up with a chimeric RNA read in our sequencing results where each side of the amplicon maps to a different transcript. We therefore performed analysis on our paired end sequencing reads to show that this does not occur at any substantial rate (see details below).

Paired-end sequencing results analysis: If cDNA sequences were able to jump to other cDNA sequences (Scenario 3), or extend on each other (Scenarios 2 or 4), in a way that led to background in our sequencing results, then we would expect to see evidence of chimeric RNA reads. To examine this possibility, we used paired end sequencing data from the from our TH^{+} DAC profiling experiment (from **Fig. 5** of the main text) and performed the following analysis on our replicates :

(1) Map R1 reads to transcriptome
(2) Map R2 reads to transcriptome
(3) Compare fraction of successful R1 and R2 mappings that match the same transcript

	Non-chimeric read pairs	Chimeric read pairs	Percentage of chimeric read pairs
**Replicate 1**	143,354	425	0.30%
**Replicate 2**	212,199	794	0.37%
**Replicate 3**	112,926	315	0.28%
**Replicate 4**	174,932	485	0.28%
**Replicate 5**	92,374	290	0.31%

Chimeric analysis for pairs where both R1 and R2 reads mapped to transcripts.

While we do not see strong evidence of jumping between mRNA transcripts in our data, we note that because we are doing tagmentation to shorten the reads and enriching specifically for the barcode side of our amplicons, evidence of such an effect might be less obvious in our sequencing data. However, based on our differential gene expression analysis showing strong enrichment of genes that were orthogonally and directly validated to localize in those regions with smFISH, we do not expect this to be confounding our results in any significant way.

Supplementary Note 2. Background blocking.

Initial tissue barcoding experiments displayed a pronounced fluorescent background on the surface of the well. We suspect that both the PDL coating and OCT mounting media (cryoprotectant) causes fluorescent DNA barcode strands to stick to the glass surface, which could lead to unwanted background in downstream amplification steps. This led us to optimize our barcode hybridization buffer to improve the tissue barcoding experiments. Specifically, we added 2 mg/ml sheared salmon sperm DNA (Invitrogen cat. no. AM9680) and 10% dextran sulfate (wt/vol, Sigma Aldrich cat. no. S4030) to the barcode hybridization solution, which significantly improved the signal-to-noise ratio of the fluorescent barcode signal (data not shown). We have updated the detailed protocols at ***lightseq.io*** for the cell culture also with this recommended change, and we highly recommend using this improved hybridization protocol in future applications.

Supplementary Note 3. Intronic mapping for retina tissue experiments.

Our standard pipeline uses the default settings of *featureCounts*[5], which maps only exons. To examine the proportion of intronic reads, we also mapped to genes (with the `-t gene` flag of *featureCounts*) rather than just exons. After deduplication, we ran our sequences that mapped to gene bodies through the RSeQC software[6] to analyze the number of intronic maps.

We found ~21-26% intron mapping across replicates for our retina cell type layer experiment, and ~25-54% intron mapping across replicates for the rare amacrine cell type experiment. These values, additional mapping statistics (e.g. for CDS exons, 5' UTR exons, 3' UTR exons), and a screenshot from Integrative Genomics Viewer (IGV)[7] of an example gene containing intronic reads are shown below. Please note that intron to exon percentages could vary more for smaller input sizes (such as the 4-8 cells per sample in the amacrine cell experiment) depending on the cut of section and focusing of the illumination.

	Replicate 1			Replicate 2			Replicate 3			Replicate 4		
	Tag count	Perc	Tags/Kb	Tag count	Perc	Tags/Kb	Tag count	Perc	Tags/Kb	Tag count	Perc	Tags/Kb
CDS Exons	1,119,726	46.70%	30.47	1,015,452	49.12%	27.63	826,975	49.11%	22.50	688,816	45.67%	18.75
5' UTR Exons	94,043	3.92%	2.67	82,050	3.97%	2.33	69,143	4.11%	1.97	64,924	4.30%	1.85
3' UTR Exons	600,361	25.04%	10.03	534,609	25.86%	8.93	419,360	24.90%	7.01	365,223	24.21%	6.10
Introns	581,880	24.27%	0.53	433,636	20.97%	0.40	366,757	21.78%	0.34	387,857	25.71%	0.36
TSS upst 10Kb	879	0.04%	0.00	766	0.04%	0.00	823	0.05%	0.00	754	0.05%	0.00
TES downst 10Kb	932	0.04%	0.00	888	0.04%	0.00	812	0.05%	0.00	784	0.05%	0.00

Exonic and intronic read breakdown per replicate for retina cell type layer experiment. (Perc = percentage, upst = upstream, downst = downstream.)

	Replicate 1			Replicate 2			Replicate 3			Replicate 4			Replicate 5		
	Tag count	Perc	Tags/Kb	Tag count	Perc	Tags/Kb	Tag count	Perc	Tags/Kb	Tag count	Perc	Tags/Kb	Tag count	Perc	Tags/Kb
CDS Exons	122,465	36.06%	3.33	143,508	25.33%	3.91	113,105	44.93%	3.08	157,061	45.74%	4.27	110,396	46.55%	3.00
5' UTR Exons	12,292	3.62%	0.35	17,606	3.11%	0.50	8,805	3.50%	0.25	13,122	3.82%	0.37	8,558	3.61%	0.24
3' UTR Exons	69,493	20.46%	1.16	97,968	17.29%	1.64	59,539	23.65%	0.99	82,924	24.15%	1.39	57,612	24.29%	0.96
Introns	135,268	39.83%	0.12	307,278	54.24%	0.28	70,192	27.88%	0.06	90,142	26.25%	0.08	60,454	25.49%	0.06
TSS upst 10Kb	35	0.01%	0.00	57	0.01%	0.00	46	0.02%	0.00	42	0.01%	0.00	49	0.02%	0.00
TES downst 10Kb	67	0.02%	0.00	121	0.02%	0.00	63	0.03%	0.00	92	0.03%	0.00	79	0.03%	0.00

Exonic and intronic read breakdown per replicate for retina rare amacrine subtype experiment. (Perc = percentage, upst = upstream, downst = downstream.)

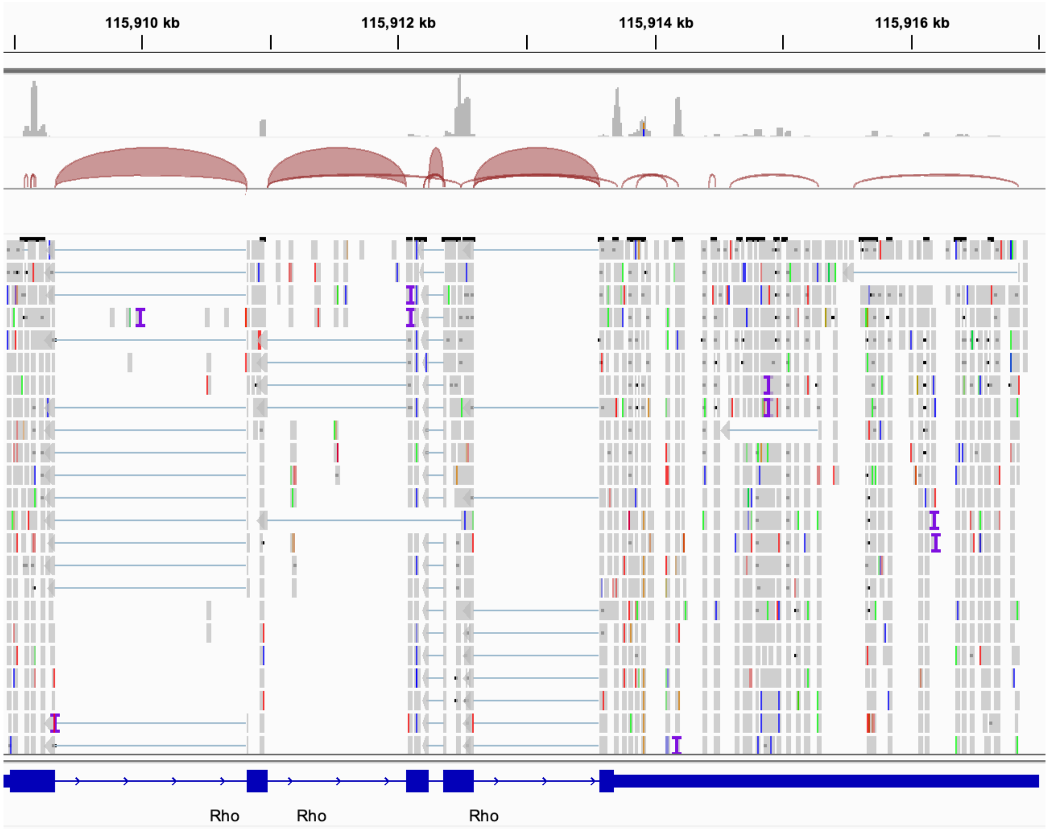

Screenshot from IGV software version 2.12.3 showing intronic, exonic, and exon-exon junction maps for the *Rho* gene from Replicate 1 of the retina cell type layer experiment.

Supplementary Note 4. Instructions for calibrating, focusing, optimizing, and selecting ROIs for DMD illumination, and important considerations for resolution and experimental design.

To achieve high-resolution light-directed barcoding for Light-Seq, the optical setup must be optimized. Here we include suggestions and tips, and we encourage users to visit ***lightseq.io*** or ***protocols.io*** platform for the most up-to-date protocols.

1. Selecting an optical system

For very fast, parallelized barcoding, we use a DMD and LED light-source. For more precise illumination, however, one could also use a laser-scanning confocal microscope (see **Extended Data Fig. 1**) or another optical system which enables focusing of 365-405 nm light to prescribed regions of the sample for selective barcoding.

There exist many off-the-shelf systems that can be used to focus UV light in 3-dimensions, but we provide detailed instructions for our optical setup below. For any new system, we recommend familiarizing yourself with any available documentation (e.g. manuals) and having a conversation with your microscope representative to get their best practices and advice. There exist many other scientific applications that require targeted light delivery with a wealth existing literature (e.g. optogenetics), and these complementary protocols can be used as a reference.

2. Optical setup overview

For all of the barcoding data from the main figure panels (Figures 2, 3, 4, and 5), our barcoding setup was an inverted Nikon Eclipse Ti-E microscope with an attached Mightex Polygon 400 DP DMD. Our UV light source was a Mightex BLS-series high-power liquid light guide coupled LED source, 365 nm, 50 W emitter focused through through a CFI plan fluor 10X objective.

The Nikon Elements software (version 4.51) is fully integrated with the control of the DMD system so that all of the DMD's functionality can be accessed through the Nikon microscope GUI. A screenshot of the Nikon interface with the Mightex widget is shown below.

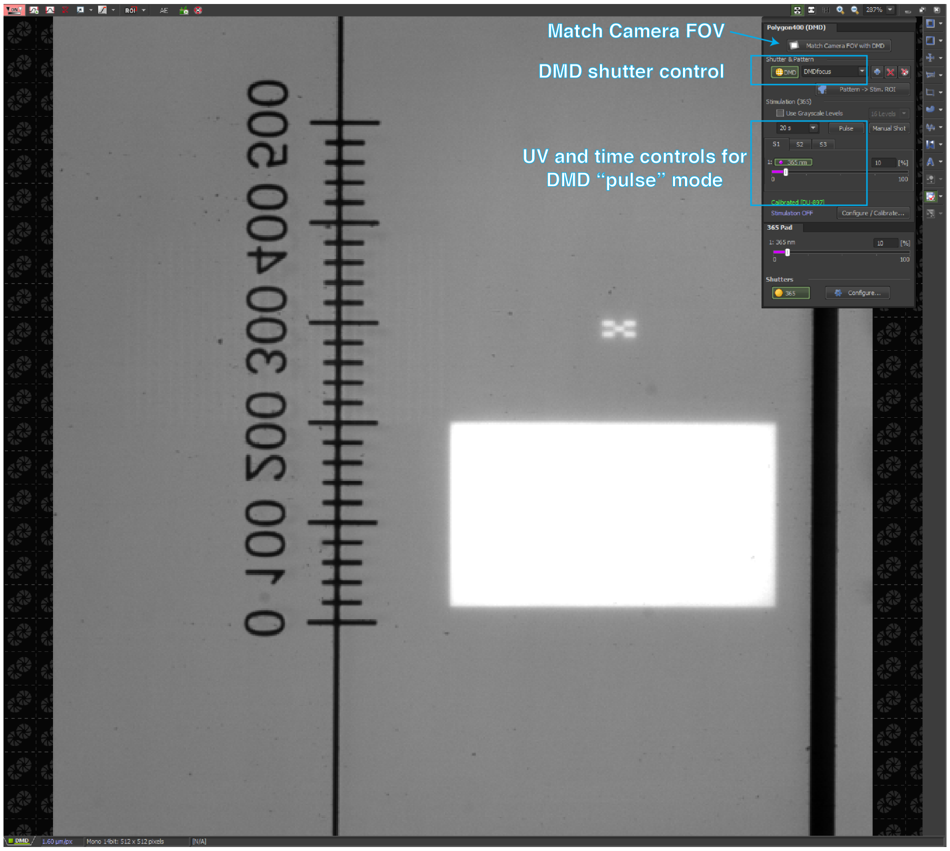

Supplementary Note Figure 1. Screenshot of the Polygon 400 widget interface. A custom UV photopattern is being projected onto a calibration slide.

Objective selection:
The optimal crosslinking wavelength for CNVK is 365 nm[8]. This is considered long-wavelength UV and therefore it is important to use a compatible objective for UV light. On the Nikon objective selector website, you can see the wavelength transmission for all available objectives. One example is given here https://www.microscope.healthcare.nikon.com/products/optics/selector/comparison/-1953. As a general rule, most Plan Fluor or Super Fluor objectives have good UV transmission, whereas Plan Apo is generally not recommended for UV.

Power density:
The recommended power density of CNVK crosslinking with 365 nm wavelength is roughly 1 W/cm^2, which is sufficient to induce near complete crosslinking (>90%) within 1 second. This power density is generally very achievable with a microscope objective as it will focus the UV power to a small point at the sample plane. However, improper focus will likely dramatically affect the UV power at the sample plane and may lead to poor crosslinking.

Other wavelengths of light:
In this manuscript, we briefly tested other wavelengths of light (405 nm) to introduce alternative wavelength options for photo-crosslinking. 405 nm is an attractive alternative as it is a widely available light source and compatible with nearly all commercial optical setups. While 405 nm is not the optimal wavelength for crosslinking, if the light source is a laser, the 405 nm light can typically be focused at a much higher power density than an LED, which potentially can compensate for the lower efficiency of crosslinking at the 405 nm wavelength.

Note that in our hands we have observed a small amount of background crosslinking (~10%) even at the 561 nm wavelength, however this typically happens over a much longer timescale (~30 min). This is an important factor to consider when using a laser light source to identify fluorescent targets prior to barcoding. In this case, we recommend taking a quick snapshot (~1 s) of the image and then drawing the barcoding ROI on the snapshot.

3. Sample setup

In addition to the optical system, sample preparation must also be optimized to ensure targeted light delivery to the regions of interest (ROIs) within the sample. For Light-Seq, we recommend sectioning tissues or plating cells on a coverslip rather than a thick glass slide. The coverslip ensures minimal disruption to the light-path and results in more accurate spatial targeting of ROIs than barcoding through a thick glass slide (e.g. Fisherbrand™ Superfrost™ Plus Microscope Slides cat. no. 12-550-15).

In this publication, we sectioned into ibidi chambered coverslips (custom order 81824); similar larger size CAT#: 80824), which have a coverslip bottom and separated plastic chambers for contained liquid exchanges. Thus, all barcoding and imaging was done through the coverslip. We recommend coating the coverslip with poly lysine to promote tissue adhesion, as many tissues will not adhere well to the untreated coverslip glass.

We have also successfully barcoded through Superfrost™ Plus Microscope Slides, which typically have a thickness of 1 mm, with our optical system outlined above. However, light refraction does occur and will impact resolution and accuracy of UV photo-crosslinking. For larger regional barcoding, the imprecision of boundaries can be tolerated, but for applications aiming to label individual cells or thin regions, we highly recommend barcoding through a coverslip.

Additional resources and suggestions for sample preparation can be found at ***lightseq.io*** and on the ***protocols.io*** platform.

4. Calibrating the DMD to the camera field of view (X, Y dimension)

Before each experiment, a short calibration should be performed to map the camera pixels to the correct location on the DMD chip. This is mostly a software correction and can be performed as follows.

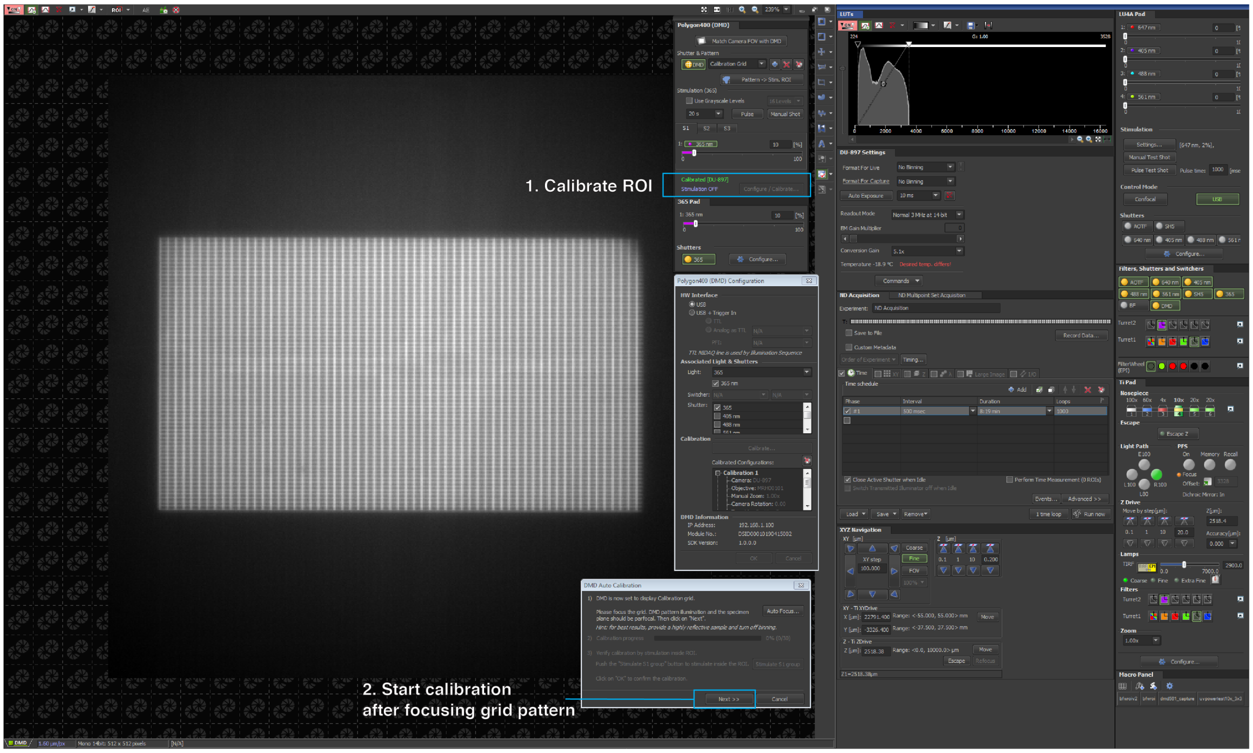

Supplementary Note Figure 2. Screenshot of the calibration widgets. The DMD will project a calibration grid photomask when performing this function.

To calibrate the DMD illumination in X/Y, press [Calibrate] button and follow the instructions on the screen. The calibration process should not take more than 30 s. Once the calibration is finished, you can test out some ROI drawings with the ROI toolbar and set it to a “stimulation region”. The term stimulation region is the notation that Mightex uses for their software interface.

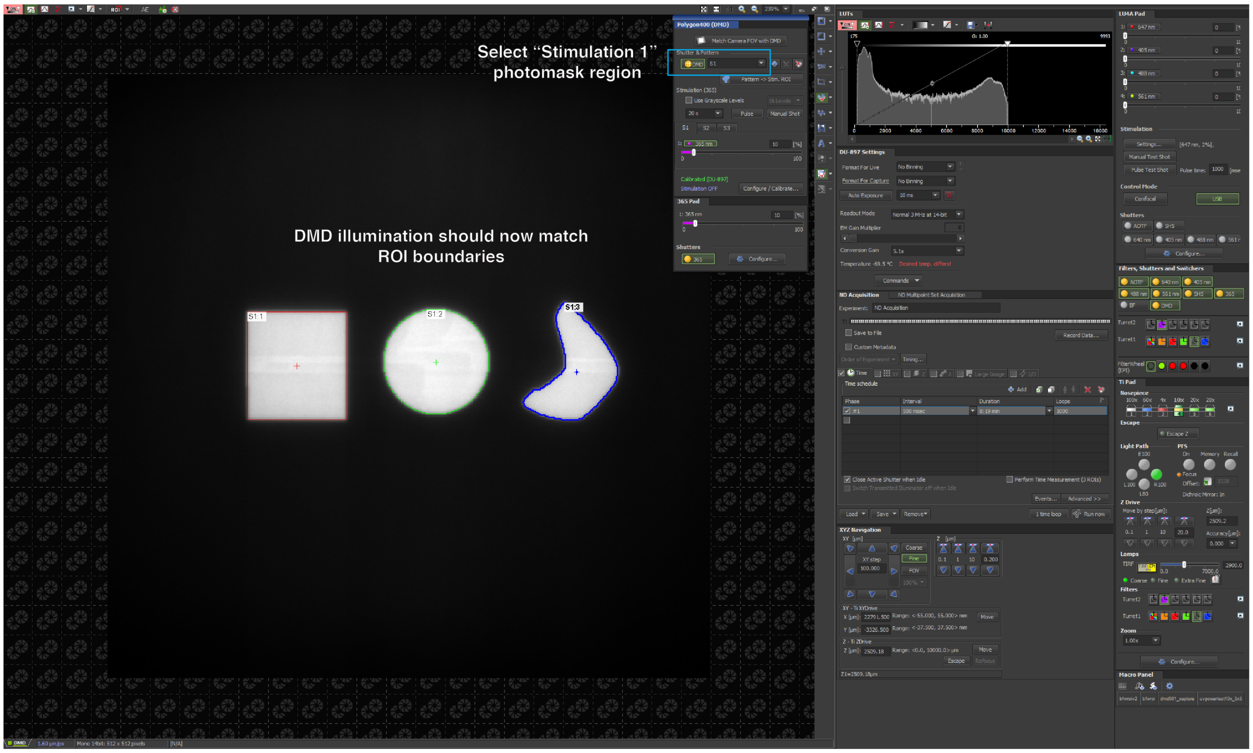

Supplementary Note Figure 3. Screenshot of the UV photomask within a stimulation region after performing the ROI calibration.

Once the DMD has been calibrated, the next step is to match the camera field-of-view (FOV) with the DMD chip. For most microscope setups, the FOV of the camera is typically larger than that of the DMD chip size, meaning that the regions near the edge of the FOV of the camera cannot be reached by the UV light. You can see this when looking at the calibration grid figure (**Supplemental Note Fig. 2**), above - the full DMD chip is used to display the calibration grid but it only occupies a fraction of the camera FOV. Once the Match Camera FOV option set to “on”, the camera FOV will automatically restrict to match the DMD chip. Example shown in a screenshot below.

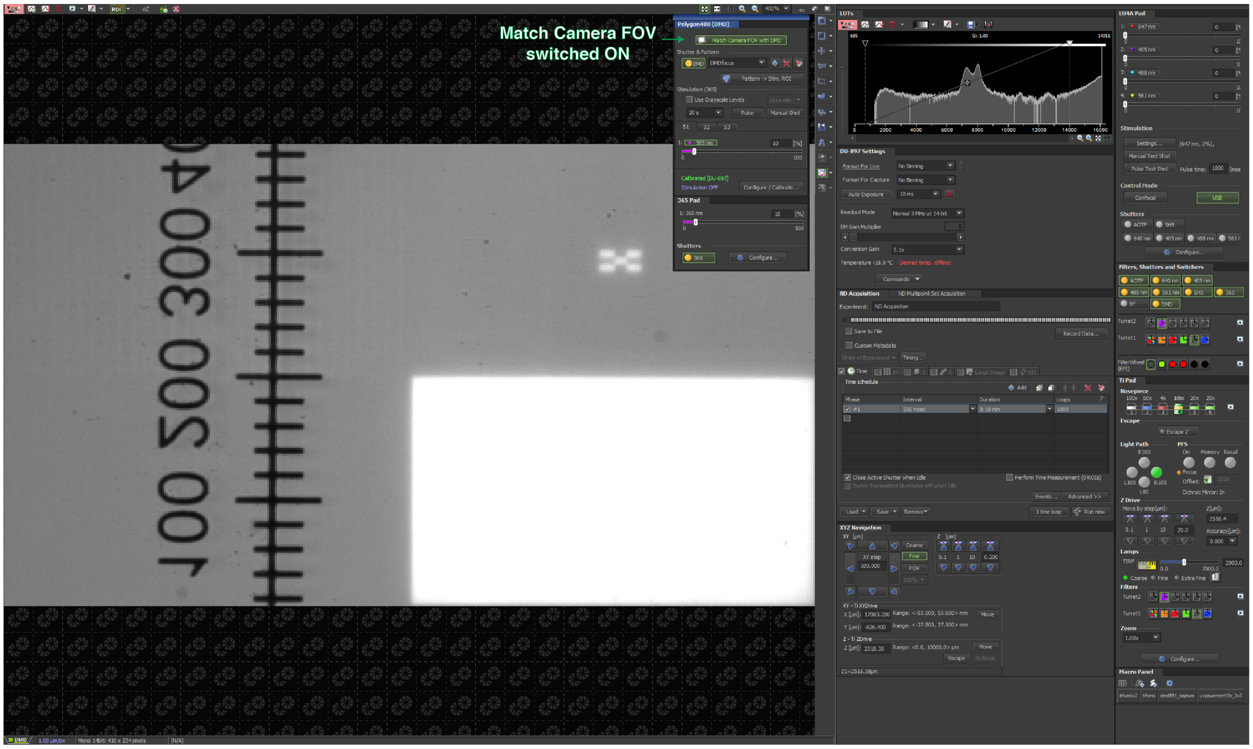

Supplementary Note Figure 4. Screenshot with the “Match Camera FOV” option turned on. Note that this image is the same region as shown in the Supplemental Note Figure 1, but the FOV is now matched to the DMD chip.

5. Calibrating the DMD focus on the sample plane (Z dimension)

In order to precisely illuminate ROIs, the UV light projected from the DMD array must be focused at the sample plane. This calibration should be performed for any new microscope set-up or sample type as differences in optical set ups can significantly affect your crosslinking efficiency. <u>For best results, we recommend repeating this process prior to **each** experiment.</u>

For the experiments performed in this publication, we calibrated the DMD focus manually to illuminate at the surface of the slide and applied small z adjustments to account for sample thickness. Figure overview is shown below with step-by-step instructions.

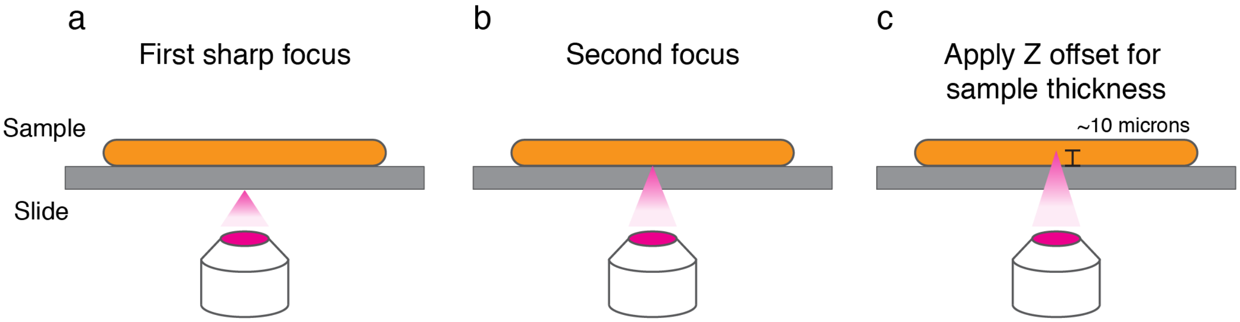

Supplementary Note Figure 5. Illustration of the different focal points

1. Place a standard microscopy slide or coverslip on the microscope.
2. Project a UV pattern onto the surface, typically a checkerboard or some other calibration pattern will be sufficient.

3. Move the objective all the way down in Z, the projected pattern should now be completely out of focus.
4. Bring the objective up slowly to reach the first "sharp" focus of the DMD pattern. (**Supplementary Note Fig. 5a**)
    a. This is the reflected light from the *bottom* of the slide, if using an air objective, this would be the air/glass interface.
    b. Note the Z position of the objective
5. Bring the objective up even more slowly until you reach a second, but much fainter focus of the DMD pattern. (**Supplementary Note Fig. 5b**)
    a. If the slide has a sample, this would be the glass/water or glass/sample interface, which will not give as sharp of a reflection.
    b. Note the difference in Z position to arrive at this second focus.
    c. You can calculate the optical path length with $n \times s$, where n is the refractive index of glass (1.51) and s is the difference in Z positions between the first and second focus. The calculated optical path length should match the thickness of the glass used.
        i. With a standard 1mm thick microscopy slide, you can expect this Z difference to be ~660 µm. 660 * 1.51 = 1000 µm.
        ii. With a No. 1.5 thick coverslip (170 µm), you can expect this Z difference to be ~111 microns. 111 * 1.51 = 170 µm.
        iii. Note that due to variations in glass thickness, or potentially chromatic aberrations introduced by the objective, the actual distance calculated for the difference in Z can vary by 10 µm or more than what is calculated above.
6. Once the DMD is focused at the surface of the slide (i.e. the glass/sample interface), you can apply an additional Z offset to account for sample thickness. (**Supplementary Note Fig. 5c**)
    a. For example, the mouse retina section was sliced at a thickness of 15-18 µm. Therefore, we added an additional 10 µm to the focal plane in order to barcode a section layer that is closer to the topical layer of the retina.
7. Once the focal plane is selected, you can engage a Perfect Focus System (on Nikon microscopes) or an equivalent focal offset system to maintain this focal plane when moving to different areas of the slide.

We recommend focusing close to the top of the tissue section, unless you are specifically targeting a region near the bottom of your sample, or if your sample is already very thin. For example, some FFPE sections are only 5 µm thick and do not necessitate any Z-offset. For the retinal layers experiment, we chose a region near the top of the tissue as we hypothesized that reagents would diffuse more readily into the top layer of the tissue and cDNA libraries would diffuse out more easily compared to the tissue-glass interface at the bottom of the sample. However, we have not extensively tested this hypothesis.

Note that this calibration may change if you switch objectives or make other changes to the optical path of the microscope.

<u>Important Notes</u>
Note 1: You may not see a sharp pattern reflected on the camera when you are crosslinking a sample. This is to be expected as the sample will not be expected to have a large refractive index change and will likely not reflect a significant amount of light back into the camera.

Note 2: Furthermore, there may also be a blurry reflection on the outside of the ROI selection, this is also to be expected as some UV light can be reflected from the *bottom* of the slide at the glass/air interface (if using an air objective). This light is only visible because it is captured by the camera but it is NOT focused on the sample plane.

Note 3: It is important that one does not adjust the DMD focus to view a "sharp" reflection once the DMD is calibrated to be at the same focal plane as the sample. This will result in either low efficiency crosslinking, due to improper focus, or a blurry out-of-ROI crosslinking.

Note 4: Some commercial DMD systems come with an "auto-focus" functionality so that the user does not have to do this calibration every experiment. This is especially useful when using higher magnification objectives (e.g. 20X or higher) as small changes in Z can significantly change the DMD focus. However, the authors have not had extensive experience with this functionality and cannot comment on how well it will fit into your experiment system. Furthermore, not all DMD systems may come with this functionality and thus it is up to the user to perform this calibration for the DMD system that they choose to use.

We recommend doing these adjustments in a separate sample area (like an adjacent well) to avoid unintended UV exposure of the valuable sample regions.

6. Optimizing illumination parameters

Suboptimal illumination parameters could have a significant impact on the illumination contrast and background crosslinking. Hence, it is critical that:

- The optical system is well-aligned to allow imaging and illumination of the same plane and field of view without aberrations.
- Illumination parameters are optimized for the wavelength, power density of the optical system at the sample level, illumination/integration time and the sample in use. The energy density that each CNVK residue receives depends on all of these factors. Although it is possible to measure the power output, it gets hard to predict the optimal parameters for different samples as the sample volume and scattering may also play a role.

Hence, we strongly suggest an empirical optimization strategy based on the fluorescence signal in ROI vs. out of ROI, before starting the actual barcoding experiment. In these experiments it would be ideal to use indirect fluorescent probes to detect the barcodes after photocrosslinking, so that the quantification is not be affected by the photobleaching of the directly labeled fluorescence barcodes during the optimization run.

Our method to find the right balance of crosslinking efficiency vs. precision involved varying both the UV power and exposure time parameters in combination using a Nikon macro that will automatically raster the stage across the well and test a single crosslinking condition per position.

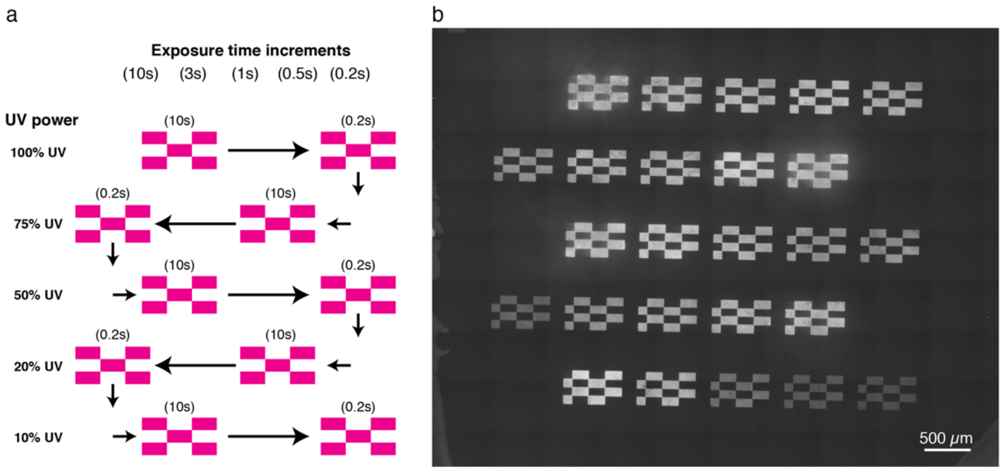

Supplementary Note Figure 6. UV power optimizations on a glass surface passivated with DNA docking strands. **(a)** Five exposure time and five UV power conditions were tested in combination, conditions were rastered in a zig-zag pattern across the well. **(b)** Fluorescent image after washing uncrosslinked strands.

From the figure above, we selected the 10% UV power for 10 seconds as a condition with a good balance between crosslinking signal and precision (as determined by the checkerboard pattern). Other conditions were also good candidates, such as 50% UV power for 1 s but were not tested extensively *in situ* for this work. Ideally this test should be done with any new sample type, within the same sample (to account for sample variability), prior to a sequencing experiment.

7. Considerations for light scattering, resolution, and experimental design

To account for light-scattering within the sample, we make ROI selections slightly smaller than the intended area barcoding area, such that the majority of cross-linking remains within the intended area (**Extended Data Fig. 6a**).

When we make a line scan over the ROI boundary, the fluorescent value decays exponentially and reaches the half value of the peak within ~3 μm and decays to background level roughly ~10 μm from the ROI boundary when the barcoding is done with a 10X air objective.

We typically use ROIs, 1-2 pixels smaller than the intended area, which translates to an ROI width of ~5 μm for most of the amacrine cells. This restrictive ROI helps to have a lower background (i.e. labeling outside of the target region) by fluorescent barcode readout (**Extended Data Fig. 6)** and despite the small spread in barcoding signal outside of the TH^+ cells, the highly enriched transcripts were well-validated by smFISH (**Fig. 5f**). It is possible that using a more precise optical setup could further increase sensitivity by limiting light-scattered crosslinking.

For finer contrast or higher resolution, higher magnification objectives and laser scanning systems would make it possible to achieve differential barcoding of adjacent objects with resolution at the level of diffraction-limit. In our previous work, we have also shown a more sophisticated application with the same chemistry and using DNA-PAINT to achieve super-resolution (single-molecule) level labeling[9]. Though this approach carries a necessary tradeoff of lower throughput and would be rather slow to perform *in situ* for spatial transcriptomics, it offers an experimental framework for future high-precision applications.

8. Selecting ROIs on Nikon software

Our DMD comes with a software add-on for Nikon Elements. Below, we show screenshots of that software during the two barcoding rounds of the cell mixing experiment (**Fig. 3**). Arbitrary photomasks (e.g. those from **Fig. 2**) can also be uploaded for illumination, and this can be combined with macros to automate the barcoding process (see **Supplementary Figs. 1-2**).

Supplementary References

1. Zhu, Y. Y., Machleder, E. M., Chenchik, A., Li, R. & Siebert, P. D. Reverse transcriptase template switching: a SMART approach for full-length cDNA library construction. *Biotechniques* **30**, 892–897 (2001).
2. Wulf, M. G. *et al.* Non-templated addition and template switching by Moloney murine leukemia virus (MMLV)-based reverse transcriptases co-occur and compete with each other. *J. Biol. Chem.* **294**, 18220–18231 (2019).
3. Turchinovich, A. *et al.* Capture and Amplification by Tailing and Switching (CATS). An ultrasensitive ligation-independent method for generation of DNA libraries for deep sequencing from picogram amounts of DNA and RNA. *RNA Biol.* **11**, 817–828 (2014).
4. Cocquet, J., Chong, A., Zhang, G. & Veitia, R. A. Reverse transcriptase template switching and false alternative transcripts. *Genomics* **88**, 127–131 (2006).
5. Liao, Y., Smyth, G. K. & Shi, W. featureCounts: an efficient general purpose program for assigning sequence reads to genomic features. *Bioinformatics* **30**, 923–930 (2014).
6. Wang, L., Wang, S. & Li, W. RSeQC: quality control of RNA-seq experiments. *Bioinformatics* **28**, 2184–2185 (2012).
7. Thorvaldsdóttir, H., Robinson, J. T. & Mesirov, J. P. Integrative Genomics Viewer (IGV): high-performance genomics data visualization and exploration. *Brief. Bioinform.* **14**, 178–192 (2013).
8. Yoshimura, Y. & Fujimoto, K. Ultrafast reversible photo-cross-linking reaction: toward in situ DNA manipulation. *Org. Lett.* **10**, 3227–3230 (2008).
9. Liu, N., Dai, M., Saka, S. K. & Yin, P. Super-resolution labelling with Action-PAINT. *Nat. Chem.* **11**, 1001–1008 (2019).
