# Supplementary material for: Light-Seq: light-directed in situ barcoding of biomolecules in fixed cells and tissues for spatially indexed sequencing
Source: Nat Methods. 2022 Oct 10;19(11):1393–402. doi: 10.1038/s41592-022-01604-1 (PMC9636025; doi:10.1038/s41592-022-01604-1)
Supplement: Supplementary file 2 — Reporting Summary [file 41592_2022_1604_MOESM2_ESM.pdf]

nature portfolio

Corresponding author(s): Jocelyn K Kishi, Constance Cepko, Sinem K Saka, Peng Yin

Last updated by author(s): 2022/06/25

Reporting Summary

Nature Portfolio wishes to improve the reproducibility of the work that we publish. This form provides structure for consistency and transparency in reporting. For further information on Nature Portfolio policies, see our Editorial Policies and the Editorial Policy Checklist.

Statistics

For all statistical analyses, confirm that the following items are present in the figure legend, table legend, main text, or Methods section.

n/a	Confirmed	
☐	☒	The exact sample size (*n*) for each experimental group/condition, given as a discrete number and unit of measurement
☐	☒	A statement on whether measurements were taken from distinct samples or whether the same sample was measured repeatedly
☐	☒	The statistical test(s) used AND whether they are one- or two-sided<br>*Only common tests should be described solely by name; describe more complex techniques in the Methods section.*
☒	☐	A description of all covariates tested
☒	☐	A description of any assumptions or corrections, such as tests of normality and adjustment for multiple comparisons
☐	☒	A full description of the statistical parameters including central tendency (e.g. means) or other basic estimates (e.g. regression coefficient) AND variation (e.g. standard deviation) or associated estimates of uncertainty (e.g. confidence intervals)
☐	☒	For null hypothesis testing, the test statistic (e.g. *F*, *t*, *r*) with confidence intervals, effect sizes, degrees of freedom and *P* value noted<br>*Give P values as exact values whenever suitable.*
☒	☐	For Bayesian analysis, information on the choice of priors and Markov chain Monte Carlo settings
☒	☐	For hierarchical and complex designs, identification of the appropriate level for tests and full reporting of outcomes
☐	☒	Estimates of effect sizes (e.g. Cohen's *d*, Pearson's *r*), indicating how they were calculated

Our web collection on statistics for biologists contains articles on many of the points above.

Software and code

Policy information about availability of computer code

Data collection	Microscope control:<br>MetaXpress software (version 6.5.3.427)<br>Nikon Elements AR 5.02 software<br>Leica LAS AF (for Leica SP5)<br>Nikon elements (version 4.51) polygon 400 module user interface for DMD control
Data analysis	Image processing:<br>Cell Profiler 4.2.1<br>FIJI (version 2.0.0-rc-69/1.52n)<br>HMS OMERO (version 5.4.6.21)<br>ASHLAR 1.12.0<br>scikit-image 0.19.0<br>Adobe illustrator (v. 2021, 2022)<br><br>Sequencing data processing and analysis:<br>HMS O2 cluster - Kernel 3.10.0<br>STAR - 2.7.9a<br>Python 3.7.5<br>PyTables 3.6.1<br>UMI-tools 1.1.1<br>samtools 1.9, 1.12<br>featureCounts (subread) 2.0.1<br>pysam 0.17.0<br>numpy 1.21.4

pandas 1.3.4
Biopython 1.79
scikit-bio 0.5.6
R 3.6.1
DESeq2 1.26.0
pheatmap 1.0.12

Plotting:
seaborn 0.11.2
matplotlib 3.5.0
ggplot2 (R 3.6.1)
Matlab 2018a

Visualization:
IGV 2.12.3

Gene body coverage and chimeric read analysis:
MacBook Pro (2021) with macOS Monterey (v12.2.1)
RSeQC v4.0.0
Python 3.10.4
R 4.1.3
UCSC gff3ToGenePred and genePredToBed scripts
GNU sed 4.2.2

A Github repository with all code may be found at: https://github.com/Harvard-MolSys-Lab/Light-Seq-Nature-Methods-2022

For manuscripts utilizing custom algorithms or software that are central to the research but not yet described in published literature, software must be made available to editors and reviewers. We strongly encourage code deposition in a community repository (e.g. GitHub). See the Nature Portfolio guidelines for submitting code & software for further information.

Data

Policy information about availability of data

All manuscripts must include a data availability statement. This statement should provide the following information, where applicable:

- Accession codes, unique identifiers, or web links for publicly available datasets
- A description of any restrictions on data availability
- For clinical datasets or third party data, please ensure that the statement adheres to our policy

Code for image analysis, sequence analysis, and differential gene analysis is accessible on GitHub at https://github.com/Harvard-MolSys-Lab/Light-Seq-Nature-Methods-2022.

Raw sequencing data is deposited online (GEO Series accession number GSE208650). Genes identified in the human-mouse cell mixing experiment are listed in Source Data Table 1 (for Extended Data Fig. 2 and Supplementary Table 2). Differentially enriched genes enriched across layers in the retina tissue experiment are listed in Source Data Table 2 (for Fig. 4). The full list of differentially enriched genes enriched between TH+ and TH- cells in the rare retinal amacrine cell tissue experiment is provided in Source Data Table 3 (for Fig. 5).

Field-specific reporting

Please select the one below that is the best fit for your research. If you are not sure, read the appropriate sections before making your selection.

☒ Life sciences ☐ Behavioural & social sciences ☐ Ecological, evolutionary & environmental sciences

For a reference copy of the document with all sections, see nature.com/documents/nr-reporting-summary-flat.pdf

Life sciences study design

All studies must disclose on these points even when the disclosure is negative.

Sample size

For cell mixing experiments ~25-30 cells were pooled from a population of ~4,500 cells per well for each of the technical replicates. The sample size was not pre-determined, but the results were validated by determining the species-specific mapping of transcriptomes from each targeted cell population. For the retinal layer experiments, 4 tissue sections were prepared from retinas dissected from 1 mouse. This number of replicates was not statistically pre-determined, but was chosen to try to recover statistically significant gene expression profiles from a small amount of starting material. 10-15% of the cells in each section were in situ barcoded for RNA-sequencing. These sample sizes are sufficient for the conclusions drawn, as the conclusions were limited to the genes which were found to be statistically differential between cell populations. For amacrine cell experiments, 4-8 TH+ cells were barcoded and pooled per section replicate (5 successful replicates in total, 1 replicate which was unsuccessful). This number was not statistically determined, but was taken as the total number of TH+ cells present within each section. Since this number fell between 4 and 8 for all sections, we ended up with a range of 4-8 TH+ cells. TH- amacrine cells transcriptomes came from a pool of roughly 500 cells per replicate (5 in total). This number was similar to the region size chosen in the prior retinal layer experiment, where we recovered consistent gene expression profiles across 4 replicates from the other cellular layers, and therefore we hypothesized that this sample size would be sufficient to find interesting and robust biological insights. These sample sizes were sufficient for the conclusions drawn, as the conclusions were limited to genes which were statistically differential between the TH+ and TH- cell populations. Of course, it is possible that larger sample sizes would result in more statistically differential genes across these populations,

	but our findings suggest that these sample sizes produced gene expression signatures consistent with orthogonal validation experiments, as covered in Figures 4 and 5.
Data exclusions	For amacrine cell experiments, 6 section replicates were prepared for barcoding, but only 5 yielded enough material during the PCR amplification of the extracted sequences and was hence sent for sequencing. Hence the sequencing data comes from 5 technical section replicates.
Replication	The cell-mixing experiment was done with 3 technical replicates where for each replicate, cells in a separate well were labeled and separately prepared and pooled for sequencing. All attempts at replication were successful. In the case of the mouse retina experiments, 4 technical replicates were performed where for each replicate a different section from the same source tissue was used to barcode and image 3 retinal layers and the extracted material for each replicates was prepared separately and later pooled for the sequencing run. All attempts at replication were successful. For evaluation and quantification of our method, multiple biological replicates were not accumulated to avoid unnecessary use of animal tissue material. For amacrine cell experiments, 6 section replicates were prepared for barcoding, but only 5 yielded enough material during the PCR amplification of the extracted sequences and was hence sent for sequencing.<br>For amacrine cell RNA-FISH validation experiments, 4 technical replicates were prepared per probe (one littermate mouse). 2 of the originally barcoded sections were revisited for validation antibody stainings after barcoding. All attempts at replication were successful.
Randomization	Randomization was not necessary for this study, since each experiment was designed to have technical replicates from the same biological specimens. For the cell culture experiment, multiple wells of the same chamber slide were simultaneously plated with human and mouse cells and processed in the same way. For the retina experiments, the sections were adjacent sections from the same retina, placed into distinct chambers on the same chamber slide, and therefore randomization was not necessary. For smFISH validation of the TH+ amacrine cell markers, the markers chosen for validation were chosen based on defined criteria (log2Fold enrichment >3 and padj<0.05) and all markers fitting this criteria were validated (with the exception of the single marker for which enough ISH probes could not be designed). These validations were performed in new animals.
Blinding	Blinding was not relevant to this study, since all technical replicates for each experiment were the same. Each replicate had multiple internal conditions (e.g. human vs. mouse cells in the same well, different cell layers in the retina), but these needed to be known to correctly and deterministically barcode each population. For analysis, blinding was also not relevant, since the sequences were separated by their spatially indexed DNA barcode for the statistical analyses.

Reporting for specific materials, systems and methods

We require information from authors about some types of materials, experimental systems and methods used in many studies. Here, indicate whether each material, system or method listed is relevant to your study. If you are not sure if a list item applies to your research, read the appropriate section before selecting a response.

Materials & experimental systems

n/a	Involved in the study
☐	☒ Antibodies
☐	☒ Eukaryotic cell lines
☒	☐ Palaeontology and archaeology
☐	☒ Animals and other organisms
☒	☐ Human research participants
☒	☐ Clinical data
☒	☐ Dual use research of concern

Methods

n/a	Involved in the study
☒	☐ ChIP-seq
☒	☐ Flow cytometry
☒	☐ MRI-based neuroimaging

Antibodies

Antibodies used	Primary antibodies used for cell samples: anti-Lamin B (sc-6216, RRID:AB_648156), mouse anti-TFAM (MA5-16148, RRID:AB_11157422), and rat anti-alpha Tubulin (MA1-80017, RRID:AB_2210201). All at 1:75 dilution from vendor-supplied stocks. Secondary antibodies (all used at 1:150 dilution, from 50% glycerol stocks prepared by 1:2 dilution of the vendor antibody with 100% glycerol): Alexa 647 anti-Mouse (Jackson ImmunoResearch 715-605-150, RRID:AB_2340862), Cy3 anti-Goat (VWR 102649-368), and Alexa 488 anti-Rat (Jackson ImmunoResearch 712-545-150, RRID:AB_2340683).<br><br>For mouse retina tissues: sheep anti-CHX10 (Exalpha X1180P, RRID:AB_2314191, diluted 1:500, rabbit anti-PAX6 (Abcam ab195045, RRID: AB_2750924, diluted 1:300), anti-TH antibody (Millipore Cat# AB152, RRID:AB_390204, diluted 1:500) and goat anti-CARTPT antibody (Thermo Fisher Scientific #PA5-47170, RRID: AB_2607700, diluted 1:20).<br><br>Secondary antibodies:<br>Donkey anti-Sheep-Alexa647, Jackson ImmunoResearch #713-605-147,RRID: AB_2340751, diluted 1:500 from 50% glycerol stock)<br>Donkey anti-Rabbit-Cy3, Jackson ImmunoResearch #711-165-152, RRID: AB_2307443, diluted 1:500 from 50% glycerol stock)<br>Donkey anti-Goat Alexa 488 secondary antibody (Jackson ImmunoResearch #705-545-003, RRID:AB_2340428, diluted 1:250)<br>Donkey anti-Rabbit Alexa647 secondary antibody (Jackson ImmunoResearch #711-605-152, RRID:AB_2492288, diluted 1:250)
Validation	All antibodies used are from commercial sources as described.<br><br>Anti-Lamin B (sc-6216, RRID:AB_648156): This antibody has been cited in 213 publications, linked at the manufacturer's website. The manufacturer shows several Western blot validations for Lamin B detection in cell lysates from multiple cell types, showing 66 kDa bands, consistent with the size of Lamin B protein.

Mouse anti-TFAM (MA5-16148, RRID:AB_11157422): According to the manufacturer's website, this antibody was validated by knockdown via siRNA, followed by Western blot.

Rat anti-alpha Tubulin (MA1-80017, RRID:AB_2210201): This antibody has been validated by the manufacturer by Western Blot in many cell types, showing 52 kDa bands corresponding to alpha Tubulin. It has also been validated for IF by the manufacturer and has been in many publications, which are sited on the product page.

Anti-TH antibody (Millipore Cat# AB152, RRID:AB_390204): According to the manufacturer's website, this antibody has been published and validated for use in ELISA, IF, IH, IH(P), IP and WB. They cite 341 publications using this antibody for detection of TH protein on their website: https://www.emdmillipore.com/US/en/product/Anti-Tyrosine-Hydroxylase-Antibody,MM_NF-AB152#anchor_REF.

Anti-CARTPT (Thermo Fisher Scientific #PA5-47170, RRID: AB_2607700): There was not validation for this antibody on the manufacturer's website, but we propose that our work in Figure 5 serves as validation of this antibody, as we showed CARTPT mRNA recovered from the TH+ population transcriptomes, and CARTPT signal by IF in the exact same cells. For further validation, we also performed RNA-FISH for Cartpt and Th in different animals and saw co-localization of CARTPT RNA with TH protein and Th RNA. Validation of this antibody is also supported by prior work from other groups demonstrating co-localization of CARTPT with TH+ amacrine cells in the retina:

S. Anna Sargsyan, P. Michael Iuvone; Cocaine- and amphetamine-regulated transcript (CART): a novel retinal neuropeptide. Invest. Ophthalmol. Vis. Sci. 2014;55(13):2641.

Gustincich S, Contini M, Gariboldi M, et al. Gene discovery in genetically labeled single dopaminergic neurons of the retina. Proc Natl Acad Sci U S A. 2004; 101: 5069–5074.

Sheep anti-CHX10 (Exalpha X1180P, RRID:AB_2314191): On the manufacturer's website, they validate specificity of this antibody to a 46 kDa protein within rat retina tissue lysate and mouse retina tissue lysate and lack of binding within rat and mouse liver. This antibody has been used in many studies where it has been validated through FISH, mouse lines, and RNA sequencing. These publications are listed on the manufacturer's website, but one such publication is:
Shekhar, K. et al. Comprehensive Classification of Retinal Bipolar Neurons by Single-Cell Transcriptomics. Cell 166, 1308-1323.e30 (2016).

Rabbit anti-PAX6 (Abcam ab195045, RRID: AB_2750924): On the manufacturer's website, they validate specificity of this antibody to detect the multiple known variants of PAX6 within mouse eyeball lysates by Western blot. The 47 kDa band is consistent with the full-length PAX6 protein while the 32 and 33 kDa bands are consistent with PAX6p32 and PAX6p33 truncations. They further show image validation in human brains, pancreas, and retina.

Eukaryotic cell lines

Policy information about cell lines

Cell line source(s)	A stable HEK293-GFP cell line (SC001) that constitutively expresses high-levels of GFP under a CMV promoter was purchased from GenTarget Inc. A mouse 3T3 cell line was purchased from ATCC (CRL-1658).
Authentication	Both cell lines had morphology and gene expression consistent with what was described for these cell lines in public databases (based on microscopy and RNA-Seq), but a formal authentication was not performed.
Mycoplasma contamination	Cell lines were not tested for mycoplasma contamination.
Commonly misidentified lines (See ICLAC register)	No commonly misidentified lines were used in this study.

Animals and other organisms

Policy information about studies involving animals; ARRIVE guidelines recommended for reporting animal research

Laboratory animals	All retina experiments were performed on tissue collected from postnatal day (P) 18 male wild-type CD1 IGS mice (Charles River). Mice were housed at Harvard Medical School at ambient temperature and humidity and a 12-hour alternating light-dark cycle.
Wild animals	No wild animals were used in this study.
Field-collected samples	No field-collected samples were used in this study.
Ethics oversight	All animal experiments were conducted in compliance with protocol IS00001679, approved by the Institutional Animal Care and Use Committee at Harvard University.

Note that full information on the approval of the study protocol must also be provided in the manuscript.
